# Novel breeding resources for the underutilised legume, lablab, based on a pangenome approach

**DOI:** 10.1270/jsbbs.24055

**Published:** 2025-02-05

**Authors:** Mark A. Chapman

**Affiliations:** 1 University of Southampton, School of Biological Sciences, Highfield Campus, Southampton, SO17 1BJ, UK

**Keywords:** genomic variation, lablab, pangenome, underutilised crop

## Abstract

Individuals across a species exhibit substantial presence-absence variation, to the extent that a reference genome from a single individual only contains a subset of the species’ genome. Cataloguing genome regions absent from a reference genome can therefore reveal novel genome regions, and some of this variation can be adaptive. In this work, existing short sequencing reads for the underutilised crop lablab (*Lablab purpureus* (L.) Sweet) were used to identify regions of the genome absent from the reference genome. Lablab is made up of two distinct gene pools, each with wild and domesticated types therefore represents an opportunity to identify gene pool-specific variation. Approximately 7.7% of the reads from eight accessions failed to map to the lablab reference genome (cv. Highworth), putatively being novel, and these were assembled and collapsed into between 735 and 12,304 contigs. Four samples were focussed on (one each wild and domesticated from each of the gene pools) and the novel contigs compared, to identify those present only in subsets of samples. Whilst the number of contigs containing sequenced with similarity to known genes in other legumes was low, there were some enriched gene ontology (GO) terms that could relate to adaptive differences between the groups and therefore contain novel genes for future lablab breeding. The approached used here has potential use in any other species.

## Introduction

Advances in genome sequencing have revealed surprising differences between individuals within species, not simply single nucleotide polymorphisms (SNPs) and insertions/deletions (indels), but also differences in gene copy number and presence/absence variants (PAVs). Whilst SNPs and indels can be identified routinely from aligning short read resequencing data to a reference genome, identifying PAVs is best done by assembling multiple individuals’ genomes and then comparing them. This substantial amount of variation that is only picked up when accurately comparing multiple genomes has led to the pangenome concept, i.e., a species’ genome is only accurately known when multiple individuals are compared. A reference genome is only a snapshot of the genome of that species, and this could compromise the ability to reliably resolve the genetic basis of adaptive traits.

The concept was first realised in bacteria where the first genome assemblies were possible, revealing that a single genome sequence represents only a fraction of the total genome of that species (Tettelin *et al.* 2005). For example, an early study of the *E. coli* pangenome revealed only 2200 genes found in all 17 isolates sequenced, but the total number of genes across all isolates was >15,000 (Rasko *et al.* 2008). Typically, genes found in all (or almost all, e.g., >95%) isolates are termed core, those in a single isolate are termed private, and those found in more than one but not all the genomes are termed dispensable.

Pangenomic investigations of plants are building traction, with some of the most widely grown, commercially important crops having pangenomes sequenced and analysed in the last few years; setting the stage for revolutionising crop breeding and improvement (Chapman *et al.* 2022, Della Coletta *et al.* 2021). There are a range of approaches employed. The optimal would be to sequence and assemble multiple reference genome to chromosome scale, which would most likely require using long-read sequencing (e.g., PacBIo or Nanopore), optical mapping (e.g., BioNano) and/or chromosome conformation capture (e.g., Hi-C) approaches to enable accurate assembly. These approaches are substantially more costly than short-read sequencing, such as those produced by Illumina, and therefore a trade-off is made between the contiguity of the genomes being generated and the number that can be produced. Different pangenome investigations are therefore unlikely to be completely comparable as the quality and number of genomes utilised will affect the proportion of core, dispensable and private genome regions identified. Some studies utilise a small number of very high-quality genome sequences, whereas others generate far more fragmented genome assemblies but for many more samples. Some recent examples from crop plants demonstrating these trade-offs include pangenomes for soybean (Liu *et al.* 2020), maize (Hufford *et al.* 2021), rice (Shang *et al.* 2022, Wang *et al.* 2018) and tomato (Gao *et al.* 2019) and are detailed below.

In the soybean and maize pangenomes, a small number of accessions (27 and 25, respectively) were assembled to high contiguity (N50 ~50MB and ~119 MB). In soybean, about 50% of the genes are core (present in at least 25 accessions), gene ontology (GO) analysis of the dispensable genes identified an enrichment of those involved in abiotic and biotic response, and a gene involved in seed glossiness was identified that was dispensable (Liu *et al.* 2020). In maize, only about 31% are core (present in 24 of 26 samples [26 = 25 new samples + 1 original reference genome]), and a genome wide association (GWA) analysis incorporating the PAVs identified 7% of associations were with PAVs only (i.e., no SNP was associated) including one PAV association for resistance to northern leaf blight (Hufford *et al.* 2021).

In one of the rice pangenome analyses and one for tomato, many more accessions were analysed (3010 and 725, respectively), and used short read data to generate accession-specific assemblies. The contiguity is expected to be much less using this approach (Jayakodi *et al.* 2021), and while not given for the rice example, the N50 for tomato was 3.2KB. In rice, >12,000 novel genes were identified that were absent from the reference, and after collapsing into gene families, 62% were core (in at least 99% of accessions) and there was GO enrichment for genes involved in regulation of immune and defence responses and ethylene metabolism in the non-core genes (Wang *et al.* 2018). In the tomato example, 351 Mb of novel genome sequence containing 4,837 non-reference genes was identified, and 82% of genes were identified as core (in >99% of accessions). Examining patterns of PAV loss and gain revealed a novel regulator of fruit flavour (Gao *et al.* 2019).

A notable example is another rice pangenome where an intermediate approach was used, where Nanopore long reads were generated for 251 rice accessions (mostly Asian rice and also a few African rice), and these assembled and analysed (Shang *et al.* 2022). This resulted in an intermediate level of contiguity (N50 ~11 Mb). Compared to the short-read rice analysis above where 62% of genes were core, this paper resolved 43% of genes as core, however slightly different definitions of core were used (99% in the former and 95% in the latter). In the Shang *et al.* (2022) analysis, a PAV containing a gene that negatively regulates Cadmium (Cd) uptake and accumulation was identified only in *japonica* rice that was almost absent from *indica* rice, and this correlates with the lower level of Cd in *japonica*.

Another approach is to identify reads from non-reference samples that do not align to the reference genome, and then assemble these *de novo*. In bread wheat unmapped reads from 18 samples were identified, merged, and assembled, and these contained ~21,600 predicted genes (Montenegro *et al.* 2017). It was estimated that about 64% of genes are core genes across the 19 samples, with the remainder being dispensable or private (Kongjaimun *et al.* 2022).

In the current work, a similar approach to that used in bread wheat was used, focussing on the underutilised legume lablab (*Lablab purpureus* (L.) Sweet). Lablab (2n = 2x = 22) is an annual or perennial autogamous species and can be dwarf or climbing in habit. Lablab is known for its drought tolerance and high protein content, serving as an insurance crop in some places (Primarily in Africa) and as a main crop in others (primarily in Asia) (Maass *et al.* 2010, Naeem *et al.* 2020, Pengelly and Maass 2001). Domesticated types are classified as subsp. *purpureus* and wild samples as subsp. *uncinatus*. Molecular data have confirmed a relatively deep split within lablab and these two gene pools differ in the number and size of seeds per pod (2 [often larger] or 4 [often smaller] seeds per pod). Domestication events have taken place independently in these two gene pools, although the locations are not clear (Kongjaimun *et al.* 2022, Liu 1996, Maass *et al.* 2017, Njaci *et al.* 2023, Robotham and Chapman 2015). The two subspecies are therefore not monophyletic, and some taxonomic revision is likely needed. Genome size differs between the two groups (465 Mb in the 2-seeded group and ~537 Mb in the 4-seeded group; Diakostefani *et al.* 2024) and crossing between the gene polls has only been reported once (Liu 1996), with other attempts failing.

Whilst lablab is underutilised relative to other legumes, there are named cultivars, primarily grown for forage in Australia and North America, and all named cultivars are derived from the 4-seeded gene pool (Liu 1996, Pengelly and Maass 2001) and Chapman (unpublished data). It is therefore anticipated that not only might there be adaptive variation in wild 4-seeded lablab that could facilitate the breeding of novel varieties (as investigated in the pangenome studies above), but also the entire 2-seeded gene pool appears untapped in this regard.

In contrast to the bread wheat work (Montenegro *et al.* 2017), here the samples were mapped separately, and then novel genome regions absent from the reference genome identified, allowing the exploration of subsets of accessions. In doing so, novel genome regions are identified which potentially could be used in future lablab breeding and improvement. Whilst only representing a small number of samples, future efforts to sequence lablab accessions can use the same approach.

## Materials and Methods

### Sequencing data and assembly

The lablab genome (cv. Highworth) and resequencing data from Njaci *et al.* (2023) were used in this work. Eight samples were focussed on initially, two each from the gene pools, i.e., wild 2-seeded (samples 21045 and 24800, both from Zambia), domesticated 2-seeded (13695 and 13704, both from Ethiopia), wild 4-seeded (21048 and 24750 from South Africa and Kenya, respectively), and domesticated 4-seeded (13688 and 14419 from Ethiopia and Zimbabwe, respectively). All accessions were initially obtained from ILRI (International Livestock Research Institute) and sequenced on an Illumina HiSeq2500. Approximately 20–30 million paired reads (after trimming as per Njaci *et al.* 2023) were utilised. NCBI-SRA accession numbers are given in the original publication.

Reads were mapped to the reference genome using bowtie2 v2.2.3 (Langmead and Salzberg 2012) and the settings *--very-sensitive-local*, utilising the *-un-conc* flag to output reads that did not map into a separate fastq file. These eight files of unmapped reads were then assembled separately *de novo* using abyss-pe v2.3.8 (Jackman *et al.* 2017) and a range of k-mer settings (36, 48, 60, 72, 84), resulting in 40 assemblies. Upon inspection, kmer settings 36 and 48 produced the largest number of contigs with the greatest total length (see Results) and so these were carried forward. The contigs from the kmer 36 and 48 settings for each sample were combined into one file, contigs shorter than 500bp removed, and merged using CAP3 VersionDate 02/10/15 (Huang and Madan 1999).

### Identifying novel genes and gene pool specific contigs

BLAST v2.14.1 (Altschul *et al.* 1990) searches against the CDS (coding sequences; taken from Phytozome [https://phytozome-next.jgi.doe.gov/]) of six legumes (peanut [530.v1], chickpea [492.v1], soybean [Wm82.a2.v1], lentil [718.v1], common bean [442.v2.1], and cowpea [540.v1.2]) were then utilised to identify putative coding regions in the contigs. A reciprocal best blast (RBB) analysis of all eight files of CAP3 merged contigs revealed almost no all-by-all RBBs, hence one sample from each of the gene pools (21045, 13695, 21048 and 13688) was used for the RBB analysis and the results mined to identify contigs present in only one, two, three or all four gene pools (but all would be absent from the reference genome due to the approach employed above). Those found only in the 2-seeded samples (13695 and 21045) and those found in the 2-seeded samples plus the wild 4-seeded sample (21048) were focussed on regarding novel adaptive genetic variation that may be useful for lablab breeding. Gene ontology (GO) analyses were carried out using agriGO v2 (Du *et al.* 2010) to determine whether any GO terms were over-represented in the different sets of novel genes. Biological Process, Molecular Function, and Cellular Component GO categories were all examined, and the reference genome used was cowpea because the hits against cowpea were the greatest in number. The minimum number of genes in each GO term was set to five. Overlap between the enriched GO terms was visualised with Venny (https://bioinfogp.cnb.csic.es/tools/venny/).

Differences between groups were examined using ANOVA if a Kolmogorov–Smirnov test determined the data to be normally distributed (at P = 0.05) or using a Kruskal Wallis test.

## Results

### Sequencing data and assembly

**Fig. 1.**
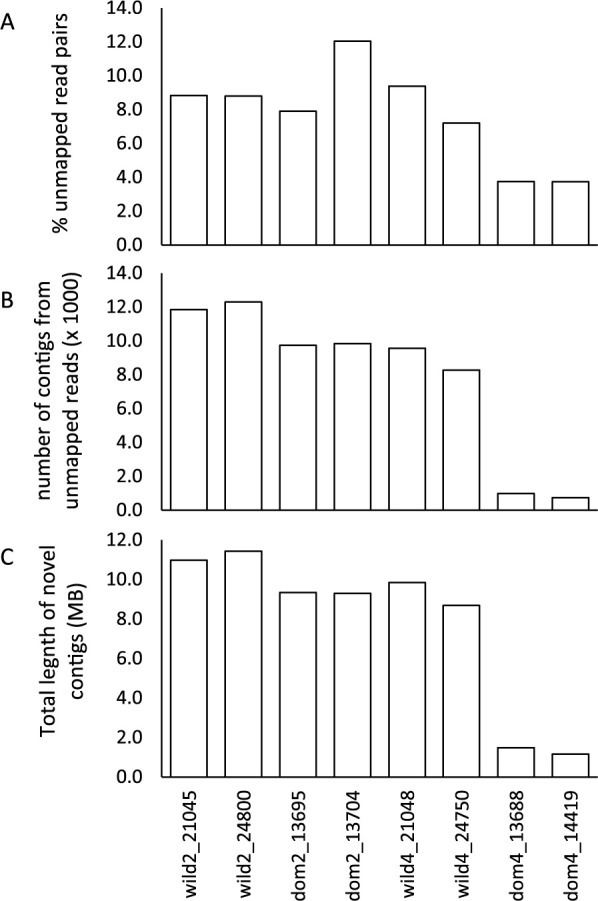
Mapping and assembly statistics for eight lablab samples mapped to the reference genome. (A) The percentage of reads that failed to map, (B) the number of contigs >500 bp (thousands) after assembling the unmapped reads, and (C) the total length of the contigs from the unmapped reads.

Of the 20–30 million reads mapped to the reference genome, between 3.73 and 12.05% of the reads failed to map (mean 7.7%). The percentage unmapped for the 4-seeded domesticated was substantially less than the other three groups (Fig. 1A). This was not significant when the four groups were treated separately (one-way ANOVA, F = 5.46, d.f. = 3, P = 0.067) but was significant when the 4-seeded domesticates were compared to all other samples (one-way ANOVA, F = 18.08, d.f. = 1, P = 0.005).

After assembling the reads, the assemblies with kmer = 36 and 48 tended to have the greatest number of contigs and the greatest total length and N50 was similar across all assemblies but tended to increase when the number of contigs decreased (Supplemental Fig. 1). Assemblies using kmer = 36 and 48 were therefore chosen for the remainder of the analysis; contigs from these assemblies were combined (per sample) and CAP3 used to merge contigs. This resulted in between 735 and 12,304 contigs per sample with significantly fewer for the domesticated 4-seeded samples than the other gene pools (one-way ANOVA, F = 197.82, d.f. = 3, P < 0.001; Fig. 1B).

Contigs tended to be small with only 25–43% being greater than 1 kb, and only 0.3–4.9% being greater than 5 kb. Contigs from the 4-seeded domesticated group tended to be longer than the other groups (Supplemental Fig. 2). Total length of the contigs from the unmapped reads varied from 1.16 MB to 11.43 MB (Fig. 1C), and was not significantly different between groups (Kruskal-Wallis Test, H = 6.00, d.f. = 3, P = 0.112); however was significantly different if 4-seeded domesticates were compared to all other samples (Kruskal-Wallis Test, H = 4.17, d.f. = 1, P = 0.046).

### Identifying novel genes and gene pool specific contigs

The number of blast hits to coding regions from six legumes was predictably higher for common bean and cowpea (mean number of blast hits >100) as they are more closely related to lablab than e.g., chickpea and lentil (Delgado-Salinas *et al.* 2011) where the mean number of blast hits was <50. While the number of novel contigs varied approximately 17-fold across samples (see above), the number of blast hits to each of the six legume species varied only three-fold (Supplemental Fig. 3); for example, mapping against cowpea the number of blast hits varied from 89 to 210.

Reciprocal blast searches were carried out for four accessions, one from each gene pool. Only 237 of the contigs were found across all four accessions which corresponded to 24.0% of the contigs from the 4-seeded domesticate sample but only 2.0–2.5% of contigs from the other three samples. Only nine of the 237 had a hit to a non-chloroplast (cp) gene.

**Fig. 2.**
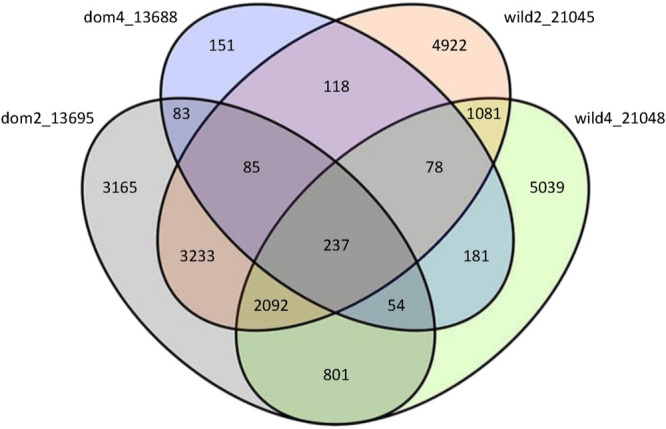
Venn diagram demonstrating the number of contigs from the unmapped reads found in one, two, three or four accessions, based on reciprocal best BLAST hits.

Between 15.3% (4-seeded domesticate) and 52.7% (4-seeded wild) of the novel contigs had no reciprocal best blast hits (i.e., were accession-specific) (Fig. 2). A gene ontology (GO) analysis of the putative genes in these accession-specific novel contigs (after removing hits to cp genes) revealed enriched GO terms for three accessions (none were enriched for the 4-seeded domesticate, which had the fewest accession-specific contigs). Comparison of the enriched GO terms for the other three accessions revealed that most terms were shared, including ‘oxidation reduction’, ‘phosphorylation’, ‘ion binding’ and ‘nucleotide binding’ (Supplemental Table 1A–1C, Supplemental Fig. 4).

A GO analysis for genes found in the contigs which were identified in both 2-seeded accessions but absent from both 4-seeded accessions (n = 30 genes from 3233 contigs) revealed an over-representation of the term ‘transferase’ and terms related to ‘nucleotide binding’ (Supplemental Table 1D). For those absent from the 4-seeded domesticate and present in the other accessions (n = 20 genes from 2092 contigs) revealed terms related to ‘oxidoreductase activity’ and ‘tetrapyrrole binding’ (Supplemental Table 1E).

## Discussion

Identifying genome regions underlying adaptive traits is an important process in crop breeding (Jayakodi *et al.* 2021, Shi *et al.* 2023), among other fields. Investigations that rely on a reference genome to associate a phenotype to a gene (or region) (Kumar *et al.* 2017) are ultimately limited by the genes present in that reference genome, as shown for virus resistance in maize (Gage *et al.* 2019). It has increasingly been shown that a single reference genome does not capture all the regions of a species’ genome. The pangenome of a species is therefore a term used to encompass the genome of a species as opposed to an individual.

Previous work in a range of crops has identified adaptive variation present in presence-absence variants (PAVs) that have been revealed using a pangenome approach and not in previous work using a single reference genome (see examples above from soybean, maize, and tomato). Other examples include a PAV for leaf senescence in rice (Qin *et al.* 2021) and silique and seed size in *Brassica napus* (Song *et al.* 2020), among others (reviewed in Zanini *et al.* 2022).

In this work, the pangenome of the underutilised legume lablab was interrogated to identify novel genome regions which could harbour genes for adaptive traits. Lablab is one of a small number of crops thought to have been domesticated more than once, with rice (Jing *et al.* 2023), barley (Morrell and Clegg 2007) and common bean (Bitocchi *et al.* 2013) being the most well-known, but also avocado (Solares *et al.* 2023), grape (Dong *et al.* 2023) and several minor crops (Meyer *et al.* 2012). A single reference genome from a crop with multiple domestications can be assumed to be missing a relatively large subset of the genomic variation in that species.

To this end, for lablab, reads from eight accessions were mapped to the reference genome and those which remained unmapped were retained, assembled, and annotated for potential coding regions. A lower percentage of reads were unmapped and fewer contigs assembled from the unmapped reads with the 4-seeded domesticated sample, which was not unexpected given the genetic similarity between the 4-seeded domesticated samples and the reference genome compared to the similarity between the other samples and the reference (Njaci *et al.* 2023).

There was only a 3-fold difference in the number of blast hits to six legume CDS libraries, despite a 17-fold difference in the number of novel contigs per accessions. This could suggest that a large portion of the novel contigs from the more distantly related samples are non-coding in nature. It is often demonstrated that dispensable genome regions in other pangenomes are enriched for repetitive DNA such as transposable elements, for example in tomato (Gao *et al.* 2019), which would support the hypothesis that repetitive regions are enriched in the novel genome regions. Another possibility is that some contigs contain genes which are not present in the other legumes, i.e., lablab-specific genes.

Genome regions novel to the 2-seeded lablab accessions (i.e., absent from the 4-seeded accessions and the reference genome) could be useful for the further domestication and breeding of lablab, especially if the genetic basis of agronomically important traits is different in the 2- and 4-seeded gene pools. For example, if seed size increase is controlled by different mechanisms in the two gene pools then there will be loci in one gene pool that could be exploited in the breeding of the other gene pool. Given that all named lablab cultivars are derived from the 4-seeded gene pool, identifying the novel genes present in the 2-seeded gene pool could be a first step in this process. In this analysis, 3233 contigs were 2-seed-specific and 2,092 contigs were 2-seed plus 4-seeded wild-specific. Within these about 1% (30 and 20, respectively) had blast hits and demonstrated an over-representation of genes involved in a few GO terms. Although hard to link some of these to adaptive phenotypes, ‘tetrapyrrole binding’ could be related to chlorophyll (Tanaka *et al.* 2011) and therefore photosynthesis, which may be worth investigating for improving photosynthesis of domesticated lablab.

To conclude, in this work a small number of lablab accessions were mined to identify novel genome regions absent from the published reference genome (from a 4-seeded gene pool accession). The total size of the novel contigs was the greatest for accessions from the 2-seeded gene pool and summed to 11.0–11.4 MB, representing about a 3% increase in the lablab genome size per accession. Only a small portion contain apparently coding regions, as is expected for a pangenome, but those that did may represent accession- and gene pool-specific adaptive pathways. This approach could be utilised in other underutilised crops where multiple high quality highly contiguous genomes are currently unavailable, but in the future may help us to identify adaptive variation in novel and underutilised crops for a future climate (Chapman *et al.* 2022).

## Author Contribution Statement

MAC carried out the work and wrote the paper.

## Supplementary Material

Supplemental Figures

Supplemental Table
