# Supplementary material for: Novel breeding resources for the underutilised legume, lablab, based on a pangenome approach
Source: Breed Sci. 2025 Feb 5;75(1):61–6. doi: 10.1270/jsbbs.24055 (PMC12203249; doi:10.1270/jsbbs.24055)
Supplement: Supplementary file 1 — Supplemental Figures [file 75_061_s1.pdf]

doi: 10.1270/jsbbs.24055

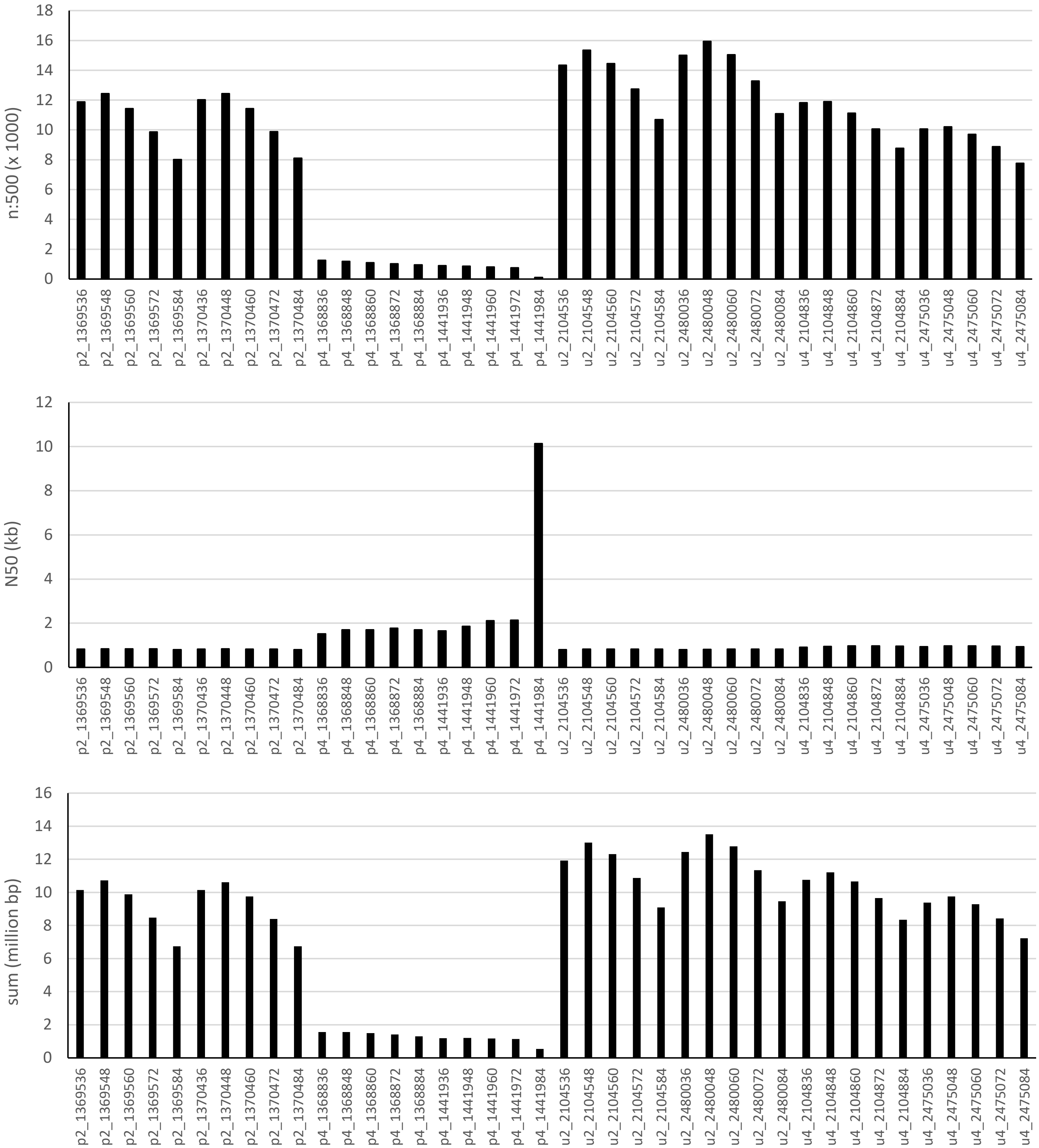

Supplemental Figure 1 – Assembly statistics for different kmer settings for the eight samples. (A) the number of contigs greater than 500bp (thousands), (B) the N50 (the size of the largest contig in the assembly such that contigs larger than that have at least 50% the bases of the assembly; in kb), and (C) the sum of the contig lengths (MB).

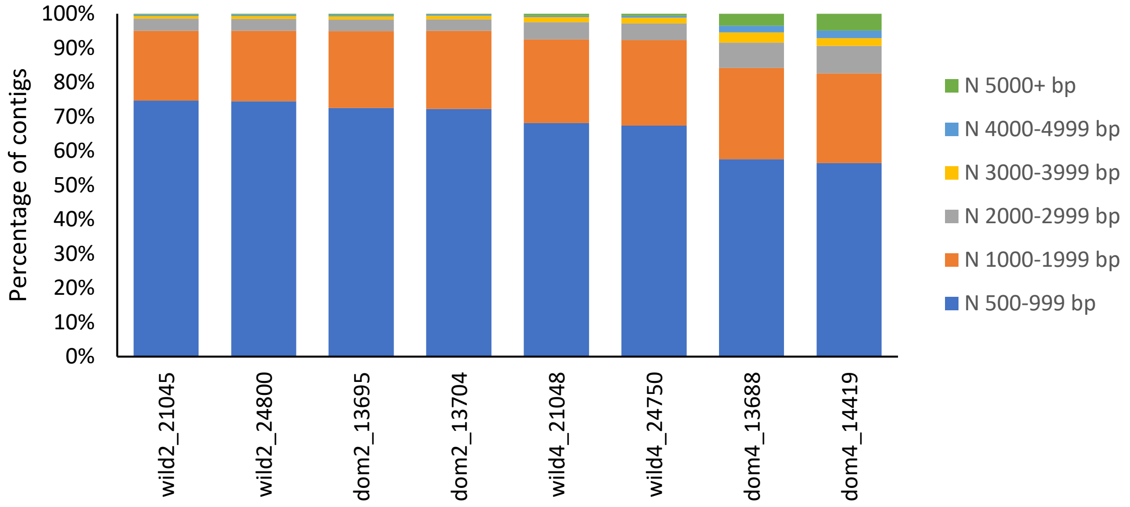

Supplemental Figure 2 – The percentage of the contigs which are of different lengths from the unmapped reads of eight samples of lablab.

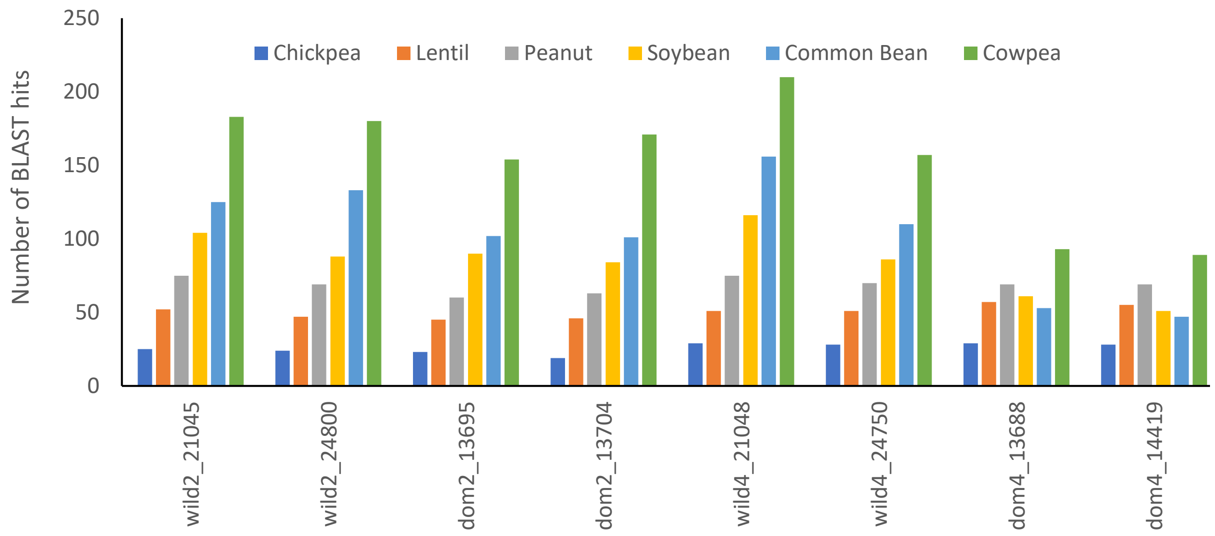

Supplemental Figure 3 – The number of BLAST hits from the novel contigs from the eight samples against six legume CDS libraries.

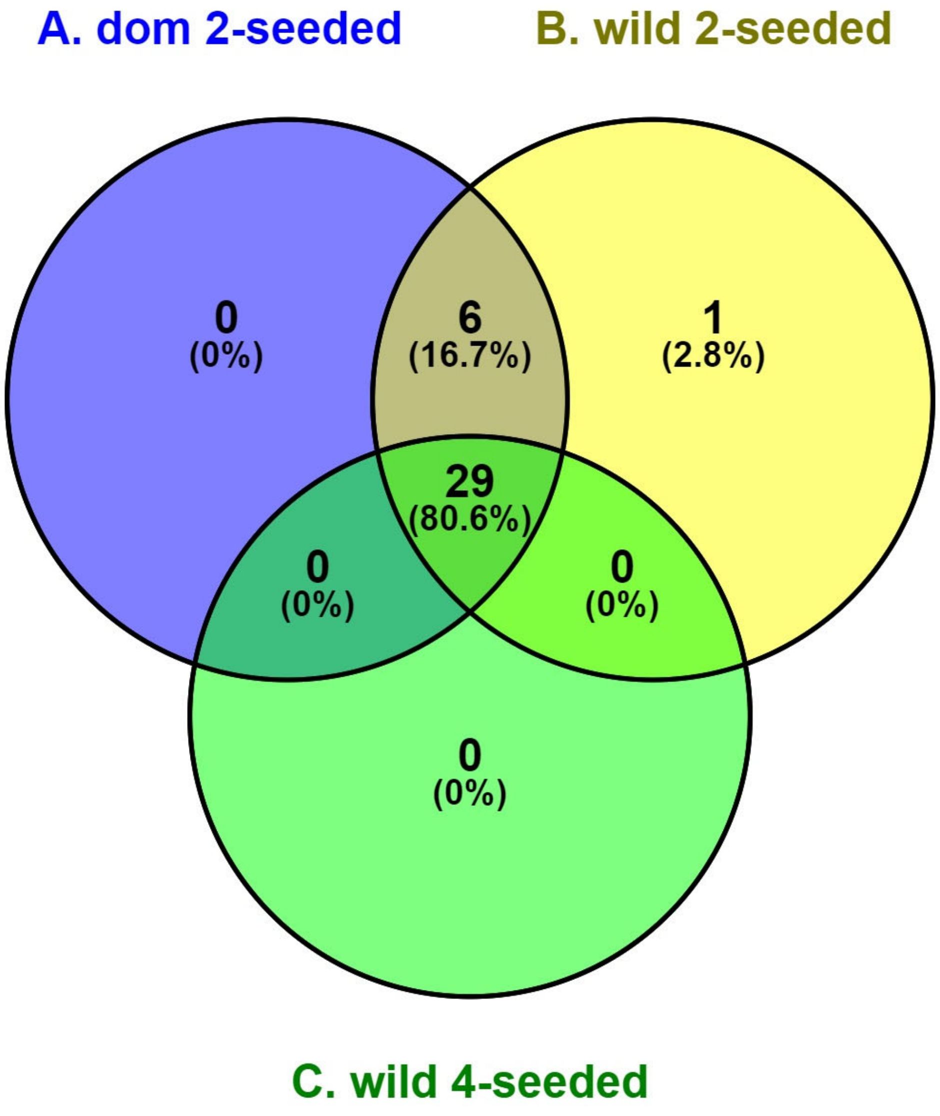

Supplemental Figure 4 – Overlap in the enriched accession-specific GO terms.
