# Supplementary material for: Novel breeding resources for the underutilised legume, lablab, based on a pangenome approach
Source: Breed Sci. 2025 Feb 5;75(1):61–6. doi: 10.1270/jsbbs.24055 (PMC12203249; doi:10.1270/jsbbs.24055)
Supplement: Supplementary file 2 — Supplemental Table [file 75_061_s2.pdf]

doi: 10.1270/jsbbs.24055

Supplemental Table 1 - Enriched GO terms related to single accessions and subsets of accessions.
Ontology is M, molecular function, B, biological process and C, cellular location.
FDR was calculated according to Yekutieli (FDR under dependency)

A. dom 2-seeded (13695) only

GO term	Ontology	Description	Number in input list	Number in BG/Ref	p-value	FDR
GO:0055114	P	oxidation reduction	11	58	9.10E-13	1.00E-10
GO:0055085	P	transmembrane transport	7	49	9.50E-08	5.20E-06
GO:0006468	P	protein amino acid phosphorylation	10	164	2.60E-07	9.60E-06
GO:0016310	P	phosphorylation	10	238	6.60E-06	0.00012
GO:0006796	P	phosphate metabolic process	11	296	6.80E-06	0.00012
GO:0006793	P	phosphorus metabolic process	11	296	6.80E-06	0.00012
GO:0043687	P	post-translational protein modification	11	376	5.90E-05	0.00092
GO:0008152	P	metabolic process	35	3002	0.00021	0.0029
GO:0006464	P	protein modification process	11	444	0.00025	0.003
GO:0043412	P	macromolecule modification	11	472	0.00041	0.0045
GO:0001882	F	nucleoside binding	18	289	1.20E-12	1.40E-10
GO:0001883	F	purine nucleoside binding	17	289	1.40E-11	5.20E-10
GO:0030554	F	adenyl nucleotide binding	17	285	1.10E-11	5.20E-10
GO:0032559	F	adenyl ribonucleotide binding	16	284	1.10E-10	3.20E-09
GO:0020037	F	heme binding	6	9	4.10E-10	9.40E-09
GO:0017076	F	purine nucleotide binding	17	425	4.20E-09	8.00E-08
GO:0032555	F	purine ribonucleotide binding	16	423	2.80E-08	4.10E-07
GO:0032553	F	ribonucleotide binding	16	423	2.80E-08	4.10E-07
GO:0000166	F	nucleotide binding	17	499	4.20E-08	5.40E-07
GO:0005506	F	iron ion binding	6	27	8.20E-08	9.40E-07
GO:0016740	F	transferase activity	20	743	9.20E-08	9.60E-07
GO:0016772	F	transferase activity, transferring phosphorus-containing groups	14	373	2.70E-07	2.60E-06
GO:0046906	F	tetrapyrrole binding	6	35	3.20E-07	2.80E-06
GO:0004672	F	protein kinase activity	10	190	9.50E-07	7.80E-06
GO:0005488	F	binding	36	2657	4.00E-06	2.90E-05
GO:0016773	F	phosphotransferase activity, alcohol group as acceptor	10	224	4.00E-06	2.90E-05
GO:0003824	F	catalytic activity	36	2750	9.40E-06	6.40E-05
GO:0005524	F	ATP binding	10	281	2.70E-05	0.00017
GO:0046914	F	transition metal ion binding	9	241	4.90E-05	0.0003
GO:0016705	F	oxidoreductase activity, acting on paired donors, with incorporation or reduction of molecular oxygen	5	55	5.50E-05	0.00031
GO:0016301	F	kinase activity	10	313	6.50E-05	0.00036
GO:0043169	F	cation binding	10	434	0.00085	0.0042
GO:0046872	F	metal ion binding	10	434	0.00085	0.0042
GO:0043167	F	ion binding	10	439	0.00092	0.0044
GO:0016491	F	oxidoreductase activity	12	612	0.001	0.0046

B. wild 2-seeded (21045) only

GO term	Ontology	Description	Number in input list	Number in BG/Ref	p-value	FDR
GO:0055114	P	oxidation reduction	11	58	5.10E-12	6.60E-10
GO:0055085	P	transmembrane transport	6	49	4.60E-06	0.0003
GO:0006468	P	protein amino acid phosphorylation	9	164	8.90E-06	0.00039
GO:0016310	P	phosphorylation	9	238	0.00015	0.0048
GO:0008152	P	metabolic process	38	3002	0.00071	0.013
GO:0006796	P	phosphate metabolic process	9	296	0.0007	0.013
GO:0006793	P	phosphorus metabolic process	9	296	0.0007	0.013
GO:0043412	P	macromolecule modification	11	472	0.0015	0.024
GO:0043687	P	post-translational protein modification	9	376	3.50E-03	4.50E-02
GO:0006464	P	protein modification process	10	444	3.20E-03	4.50E-02
GO:0020037	F	heme binding	7	9	1.70E-11	2.20E-09
GO:0001883	F	purine nucleoside binding	16	289	1.60E-09	4.10E-08
GO:0001882	F	nucleoside binding	16	289	1.60E-09	4.10E-08
GO:0030554	F	adenyl nucleotide binding	16	285	1.30E-09	4.10E-08
GO:0032559	F	adenyl ribonucleotide binding	16	284	1.20E-09	4.10E-08
GO:0005506	F	iron ion binding	7	27	7.20E-09	1.60E-07
GO:0046906	F	tetrapyrrole binding	7	35	3.40E-08	6.40E-07
GO:0016740	F	transferase activity	21	743	3.10E-07	3.60E-06
GO:0017076	F	purine nucleotide binding	16	425	2.80E-07	3.60E-06
GO:0032555	F	purine ribonucleotide binding	16	423	2.70E-07	3.60E-06
GO:0032553	F	ribonucleotide binding	16	423	2.70E-07	3.60E-06
GO:0000166	F	nucleotide binding	17	499	4.40E-07	4.80E-06
GO:0005488	F	binding	41	2657	1.80E-06	1.80E-05
GO:0003824	F	catalytic activity	41	2750	4.60E-06	4.30E-05
GO:0016705	F	oxidoreductase activity, acting on paired donors, with incorporation or reduction of molecular oxygen	6	55	8.50E-06	7.30E-05
GO:0004672	F	protein kinase activity	9	190	2.70E-05	0.00022

GO:0016772	F	transferase activity, transferring phosphorus-containing groups	12	373	4.90E-05	0.00037
GO:0016773	F	phosphotransferase activity, alcohol group as acceptor	9	224	9.40E-05	0.00068
GO:0005524	F	ATP binding	9	281	0.00048	0.0033
GO:0046914	F	transition metal ion binding	8	241	0.00081	0.0053
GO:0016301	F	kinase activity	9	313	0.001	0.0063
GO:0043169	F	cation binding	10	434	0.0027	0.015
GO:0046872	F	metal ion binding	10	434	0.0027	0.015
GO:0016757	F	transferase activity, transferring glycosyl groups	5	115	0.0027	0.015
GO:0043167	F	ion binding	10	439	0.0029	0.015
GO:0016491	F	oxidoreductase activity	12	612	0.0037	0.019

C. wild 4-seeded (21048) only

GO term	Ontology	Description	Number in input list	Number in BG/Ref	p-value	FDR
GO:0006468	P	protein amino acid phosphorylation	13	164	9.30E-09	7.00E-07
GO:0055114	P	oxidation reduction	9	58	1.10E-08	7.00E-07
GO:0016310	P	phosphorylation	13	238	5.70E-07	2.50E-05
GO:0006796	P	phosphate metabolic process	13	296	5.80E-06	0.00015
GO:0006793	P	phosphorus metabolic process	13	296	5.80E-06	0.00015
GO:0043687	P	post-translational protein modification	13	376	6.60E-05	0.0014
GO:0006464	P	protein modification process	14	444	8.90E-05	0.0016
GO:0043412	P	macromolecule modification	14	472	0.00017	0.0027
GO:0008152	P	metabolic process	44	3002	0.00049	0.007
GO:0001883	F	purine nucleoside binding	24	289	5.50E-16	1.70E-14
GO:0001882	F	nucleoside binding	24	289	5.50E-16	1.70E-14
GO:0030554	F	adenyl nucleotide binding	24	285	4.10E-16	1.70E-14
GO:0032559	F	adenyl ribonucleotide binding	23	284	4.20E-15	9.50E-14
GO:0017076	F	purine nucleotide binding	24	425	1.70E-12	3.00E-11
GO:0032555	F	purine ribonucleotide binding	23	423	1.10E-11	1.50E-10
GO:0032553	F	ribonucleotide binding	23	423	1.10E-11	1.50E-10
GO:0000166	F	nucleotide binding	24	499	4.30E-11	4.90E-10
GO:0004672	F	protein kinase activity	13	190	4.80E-08	4.80E-07
GO:0005488	F	binding	49	2657	1.10E-07	1.00E-06
GO:0016773	F	phosphotransferase activity, alcohol group as acceptor	13	224	2.90E-07	2.40E-06
GO:0016740	F	transferase activity	21	743	5.80E-06	4.40E-05
GO:0016301	F	kinase activity	13	313	1.00E-05	7.20E-05
GO:0016772	F	transferase activity, transferring phosphorus-containing groups	14	373	1.40E-05	9.00E-05
GO:0005524	F	ATP binding	11	281	8.80E-05	0.00053
GO:0046914	F	transition metal ion binding	9	241	5.60E-04	0.0032
GO:0003824	F	catalytic activity	40	2750	0.0014	0.0072
GO:0043169	F	cation binding	11	434	0.003	0.014
GO:0046872	F	metal ion binding	11	434	0.003	0.014
GO:0043167	F	ion binding	11	439	0.0033	0.015

D. 2seeded only (13695+21045)

GO term	Ontology	Description	Number in input list	Number in BG/Ref	p-value	FDR
GO:0030554	F	adenyl nucleotide binding	8	285	4.00E-08	6.20E-07
GO:0001883	F	purine nucleoside binding	8	289	4.50E-08	6.20E-07
GO:0001882	F	nucleoside binding	8	289	4.50E-08	6.20E-07
GO:0032559	F	adenyl ribonucleotide binding	7	284	8.50E-07	7.10E-06
GO:0017076	F	purine nucleotide binding	8	425	8.20E-07	7.10E-06
GO:0000166	F	nucleotide binding	8	499	2.70E-06	1.90E-05
GO:0016740	F	transferase activity	9	743	5.00E-06	2.90E-05
GO:0032553	F	ribonucleotide binding	7	423	1.20E-05	5.30E-05
GO:0032555	F	purine ribonucleotide binding	7	423	1.20E-05	5.30E-05
GO:0016772	F	transferase activity, transferring phosphorus-containing groups	6	373	6.80E-05	2.80E-04
GO:0005524	F	ATP binding	5	281	2.00E-04	7.50E-04
GO:0003824	F	catalytic activity	11	2750	7.80E-03	2.70E-02

E. 2seeded and wild4seeded only (13695+21045+21048)

GO term	Ontology	Description	Number in input list	Number in BG/Ref	p-value	FDR
GO:0055114	P	oxidation reduction	5	58	8.70E-08	4.80E-07
GO:0020037	F	heme binding	5	9	2.60E-11	7.60E-10
GO:0005506	F	iron ion binding	5	27	2.60E-09	3.80E-08
GO:0046906	F	tetrapyrrole binding	5	35	8.30E-09	8.10E-08
GO:0046914	F	transition metal ion binding	5	241	7.20E-05	0.00053
GO:0016491	F	oxidoreductase activity	6	612	6.90E-04	0.004
GO:0043169	F	cation binding	5	434	0.0011	0.0041
GO:0043167	F	ion binding	5	439	0.0011	0.0041
GO:0046872	F	metal ion binding	5	434	0.0011	0.0041
GO:0003824	F	catalytic activity	11	2750	0.0042	0.014
GO:0005488	F	binding	10	2657	0.012	0.036
